# Supplementary material for: Several methods for assessing research waste in reviews with a systematic search: a scoping review
Source: PeerJ. 2024 Nov 18;12:e18466. doi: 10.7717/peerj.18466 (PMC11580664; doi:10.7717/peerj.18466)
Supplement: Supplemental Information 1 — The findings aim to assist researchers in choosing suitable methodologies, thus contributing to the conversation on enhancing research efficiency. [file peerj-12-18466-s001.pdf]

How do systematic reviews, scoping reviews, and overviews of reviews examine research waste?

METHODS

SCOPING REVIEW
Of the databases
Pubmed and Embase

RESULTS

93 Reviews with a systematic search were included

49 methods + **33** descriptive

54% of the reviews examine for methodological research waste

45% of the reviews examine for underreported research waste

IMPLICATIONS

This review guides researchers in selecting methodologies and contributes to the ongoing discourse on optimizing research efficiency

PeerJ
Several methods for assessment of research waste in systematic reviews: a scoping review
PeerJ DOI:

This is an open access graphic distributed under the terms of the Creative Commons Attribution License.
